# Heparin enables the reliable detection of endotoxin in human serum samples using the Limulus amebocyte lysate assay

**DOI:** 10.1038/s41598-024-52735-8

**Published:** 2024-01-29

**Authors:** Stephan Harm, Claudia Schildböck, Denisa Cont, Viktoria Weber

**Affiliations:** 1 Department for Biomedical Research, University for Continuing Education Krems, Krems, Austria; 2 Department of Pharmacology, Physiology, and Microbiology, Karl Landsteiner University, Krems, Austria

**Keywords:** Immunology, Microbiology, Health care

## Abstract

The determination of lipopolysaccharide (endotoxin) in serum or plasma samples using Limulus amebocyte lysate (LAL)-based assays is currently not sufficiently reliable in clinical diagnostics due to numerous interfering factors that strongly reduce the recovery of LPS in clinical samples. The specific plasma components responsible for the endotoxin neutralizing capacity of human blood remain to be identified. There are indications that certain endotoxin-neutralizing proteins or peptides, which are part of the host defense peptides/proteins of the innate immune system may be responsible for this effect. Based on our finding that several antimicrobial peptides can be neutralized by the polyanion heparin, we developed a heparin-containing diluent for serum and plasma samples, which enables reliable quantification of LPS measurement in clinical samples using the LAL assay. In a preclinical study involving 40 donors, this improved protocol yielded an over eightfold increase in LPS recovery in serum samples, as compared to the standard protocol. This modified protocol of sample pretreatment could make LPS measurement a valuable tool in medical diagnostics.

## Introduction

Lipopolysaccharides (LPS), also termed endotoxins, are main components of the outer membrane of Gram-negative bacteria. They are glycolipids consisting of three domains, the structurally conserved hydrophobic lipid A, the core oligosaccharide, which is linked to glucosamine residues of lipid A, and the O antigen, a repeating oligosaccharide comprising two to eight sugars. LPS is released into the environment during cell growth, cell division, and cell death^1^. Its depletion or chemical destruction on surfaces or in solutions is a major challenge due to its high chemical and thermal stability. The decomposition of LPS requires exposure to temperature of 250 °C for 30 min or 180 °C for more than 3 h^2^.

As primary bacterial component encountered by the immune system of the host, LPS has a major role in bacterial pathogenicity. It acts as a pathogen-associated molecular pattern (PAMP) with toll-like receptor 4 (TLR4) as primary receptor^3^. Its entry into the bloodstream triggers the release of cytokines by monocytes and neutrophils, as well as the production of co-stimulatory molecules for the activation of the adaptive immune response^4^, and can lead to life-threatening conditions, such as sepsis. Therefore, control for endotoxin contamination of intravenously administered drugs is crucial, and maximum LPS intake of 5 Endotoxin Units (EU) per kg body weight and hour is specified in all pharmacopoeias for intravenous application of pharmaceutical and biological products^5^. Testing for putative endotoxin contamination is most commonly performed using the Limulus amebocyte lysate (LAL) assay^6–9^. LAL assays have been used for over 30 years to detect endotoxin in the quality assurance of injectable drugs and medical devices and have largely replaced the rabbit pyrogen test. They are based on using an extract of horseshoe crab hemolymph, which coagulates rapidly in the presence of endotoxin. The European Pharmacopoeia additionally describes a method based on the use of recombinant factor C, the primer of the LAL cascade^10^. The results of LAL-based LPS detection methods are expressed in EU/ml and refer to the endotoxin standards used in the test. The LAL assay can be affected by various compounds, leading to either false-positive or false-negative results. Substances that interfere with the test include divalent cations, chelating agents, surfactants, acids, antibiotics, and alcohols^11–13^. The phenomenon of endotoxin masking is caused by cationic peptides and proteins, which bind to LPS and hinder its reaction with the LAL lysate, resulting in low endotoxin recovery^14^. In particular, the application of the LAL assay for the quantification of LPS in human blood or plasma has been significantly hindered by the presence of inhibitors^15^. To overcome these interferences, several approaches to plasma pretreatment have been described, including a combination of sample dilution with pyrogen-free water and heat treatment^16^. Still, the required recovery rate of at least 50% of added LPS cannot be reliably achieved, and therefore, application of the LAL assay to detect endotoxins in the circulation has not been successful so far^17^.

Three groups of factors interfering with the LAL assay in human blood have been described^17^. Serine proteases including trypsin, factor X, and α-thrombin, can hydrolyze chromogenic substrates used in the LAL assay, leading to false-positive results. The second group comprises serine protease inhibitors, such as trypsin inhibitor and antithrombin III, which can lead to false-negative results by inhibiting LAL clotting enzymes. The third group of inhibitors are substances that directly bind to LPS and thus inhibit the activation of the LAL coagulation cascade. These substances include amphiphilic and mostly cationic polypeptides called antimicrobial peptides (AMP). While the interfering factors of the first two groups are commonly inactivated by the recommended dilution and heat treatment (75 °C, 15 min), the heat-tolerant AMPs remain present as interfering factors in the plasma samples. AMPs are important components of the innate immune system and are released during infection mainly by leukocytes, but also by platelets^18,19^. The best-known representatives of blood derived antimicrobial peptides and proteins are cationic peptide-18 (CAP-18), heparin-binding protein, platelet factor 4, lactoferrin and bactericidal/permeability-increasing protein^17^. Due to their cationic nature, they bind to the anionic lipid A part of the LPS molecule via electrostatic interactions. Their binding to LPS disrupts the outer membrane of Gram-negative bacteria, resulting in antimicrobial activity.

We have previously suggested that LPS-binding AMPs present in blood samples can neutralize LPS and thus result in low endotoxin recovery^20^. Recent studies have provided evidence that AMPs can be bound and neutralized by the polyanionic anticoagulant heparin^21,22^. Based on these findings, we describe a heparin-containing dilution reagent for plasma samples to eliminate LPS masking and suggest a modified protocol for the LAL assay to overcome its inhibition by AMPs and to allow for the reliable detection of LPS in plasma samples.

## Results

### Endotoxin neutralizing capacity

**Figure 1:**
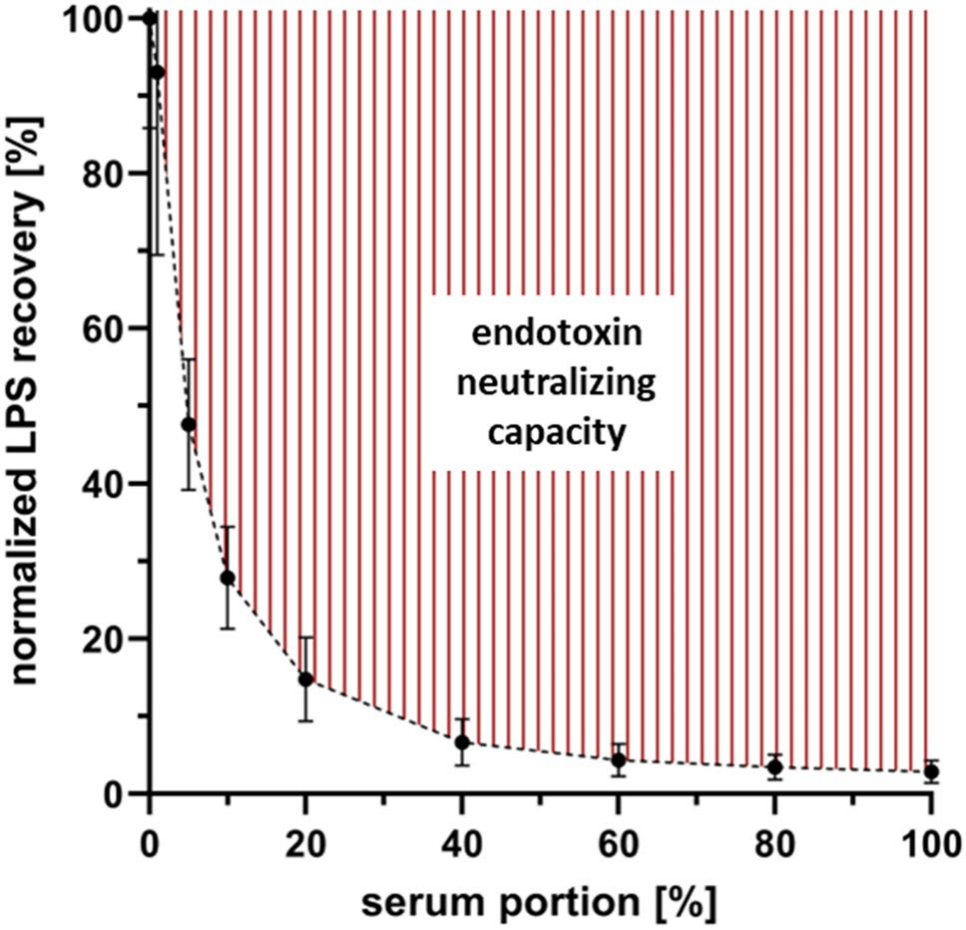
Endotoxin neutralizing capacity. Mean endotoxin neutralizing capacity (ENC) ± SD in sera from healthy donors spiked with 50 ng/ml LPS from *E. coli* (n = 6).

To determine the endotoxin neutralizing capacity (ENC) of freshly collected serum samples, a dilution series of LPS-spiked serum with physiological saline was performed. Serial dilution of serum samples resulted in increasing recovery of LPS. The recovery was 35 ± 5 times higher in aqueous solution as compared to undiluted serum. If the endotoxin value measured in aqueous solution without serum is set to 100%, the normalized LPS recovery with 1% serum content was 93 ± 24%. At a serum content of 5% (1:20 dilution), the LPS recovery was 48 ± 8% as compared to the control (without serum), indicating the presence of LPS-masking substances in human serum that reduce the sensitivity of the LAL assay. The dilution of serum samples did not result in a linear increase in LPS recovery, since even a low serum content caused significantly lower LPS recovery. The area above the curve (Fig. 1) corresponds to the ENC of human serum samples.

### Influence of heparin on endotoxin recovery in human serum

**Figure 2:**
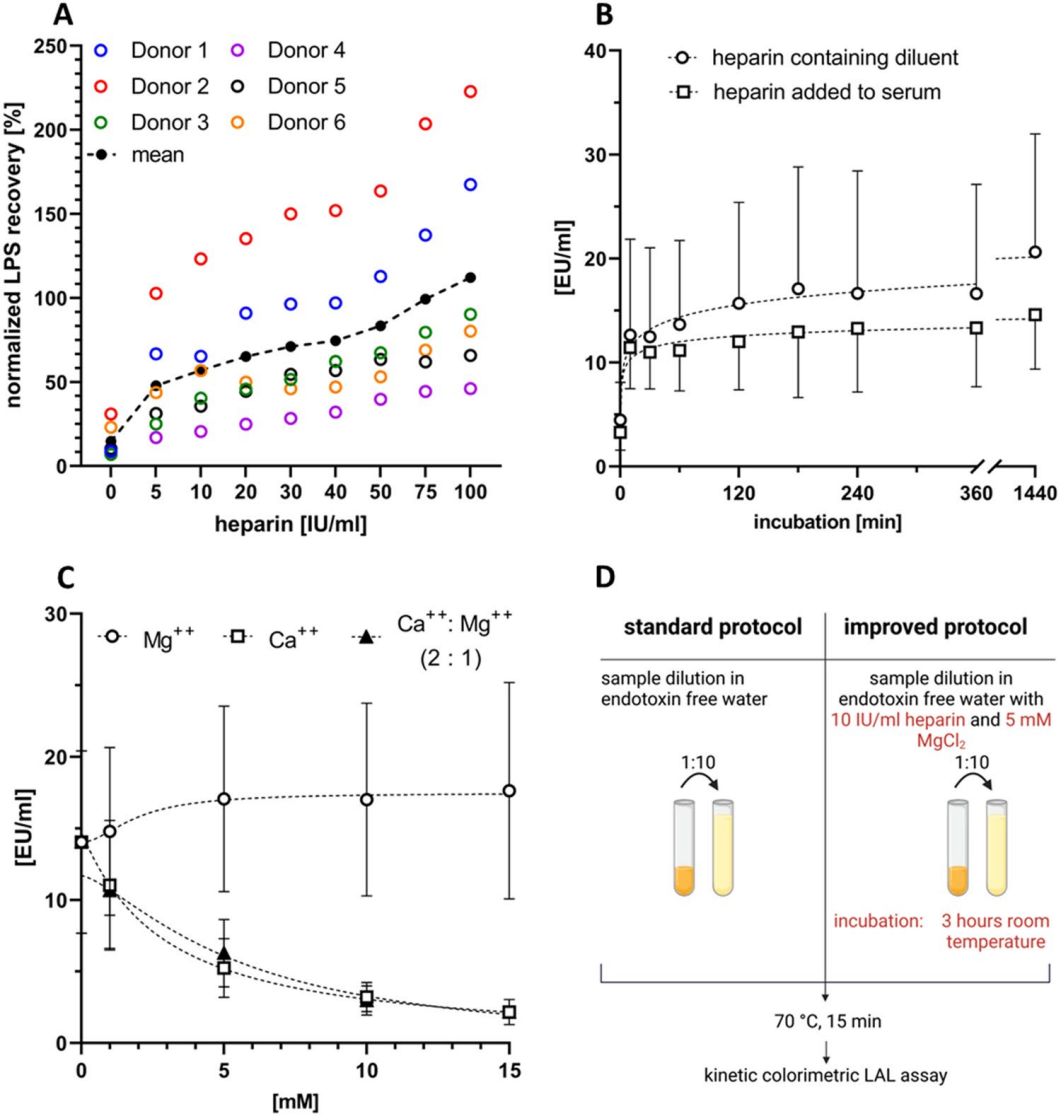
Parameters influencing LPS quantification. (**A**) LPS recovery depending on heparin concentration. Serum was spiked with increasing heparin concentrations and spiked with 50 ng/ml LPS and incubated overnight. The normalized endotoxin recovery in percent to water samples of all donors and the mean ± SD are shown (n = 6). (**B**) Influence of exposure time to heparin. Serum was spiked with 50 ng/ml LPS and incubated overnight. After incubation, each serum was once directly spiked with 100 IU/ml heparin (“heparin added to serum”) and once diluted 1:10 with heparin-containing pyrogen-free water (10 IU/ml, “heparin-containing diluent”). All samples were incubated at room temperature and analyzed with the standard LAL assay protocol. The mean ± SD is shown (n = 6). (**C**) Influence of divalent cations (Ca^++^, Mg^++^) on LPS recovery. Serum was spiked with 50 ng/ml LPS and incubated overnight. The spiked sera were mixed with heparin-containing diluent (10 IU/ml) and different Mg ^+  +^ and Ca ^++^ concentrations at a ratio of 1:10, incubated for three hours and analyzed with the standard LAL assay protocol. The mean ± SD is shown (n = 5). (**D**) Standard *vs*. improved LAL assay protocol. Based on the data shown in panels (**A**–**C**), the improved protocol for LPS quantification in plasma samples was developed.

To determine the influence of heparin on LPS recovery and to assess a potential donor dependence, sera from six different donors were spiked with 50 ng/mL LPS and increasing amounts (5–100 IU/ml) of heparin. To assess the normalized recovery of endotoxin levels in serum compared to aqueous samples, endotoxin free water was spiked with 50 ng/ml LPS and incubated overnight like the serum samples. The normalized recovery of LPS increased with increasing heparin concentration (15 ± 10% for serum without heparin *vs.* 48 ± 32% for serum containing 5 IU/ml heparin *vs*. 112 ± 68% for serum with 100 IU/ml heparin; Fig. 2A).

Next, we compared the effect of direct addition of heparin to the serum samples to the addition of heparin in the sample diluent. When heparin was added directly to serum samples or used in the diluent, an incubation of 24 h was required to achieve maximum unmasking of LPS. The recovery of LPS was significantly higher (p = 0.0005, paired t test) when a heparin-containing diluent was added compared to the direct addition of heparin in serum. Since the difference between 3 and 24 h of incubation does not result in a large change in endotoxin recovery, we decided to use a 3-h incubation period for further endotoxin testing (Fig. 2B).

When assessing the impact of divalent cations (Ca^++^, Mg^++^) on LPS quantification, we found that the LPS recovery reached its maximum in the presence of 5 mM Mg^++^, whereas the addition of Ca^++^ or a combination of Ca^++^ and Mg^++^ at a 2:1 ratio resulted in decreased LPS recovery (Fig. 2C). For all further experiments, we therefore utilized a solution of pyrogen-free water containing 5 mM Mg^++^ and 10 IU/ml heparin to perform a 1:10 dilution of the serum samples (improved protocol, Fig. 2D).

### Linearity of LAL analysis with the improved sample pretreatment

**Figure 3:**
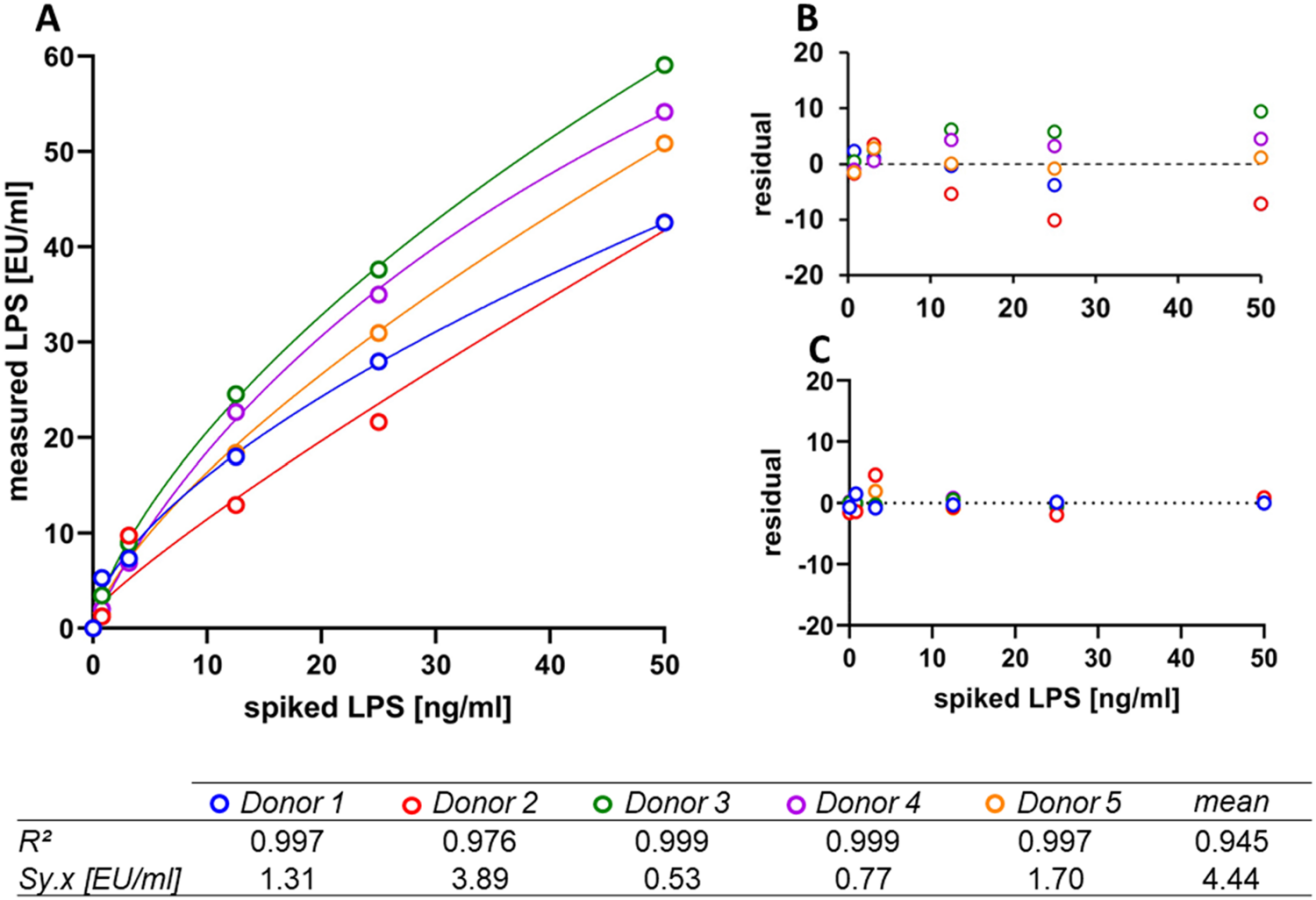
Linearity of the improved protocol. (**A**) Correlation between spiked and measured LPS values in human serum (n = 5) using the improved protocol for LAL analysis. (**B**) Residual plot (Sy.x) of mean was compared to (**C**) individual residual plots of each donor. Correlation (R^2^) of measured and spiked endotoxin levels of each donor and mean were calculated (n = 5).

Since most LAL assays are validated for quantifying LPS in a range of 0 to 50 EU/ml, we examined the linearity of the improved protocol within this range. We found that the correlation between spiked LPS (ng/ml) and measured LPS (EU/mL) in human serum of all measurements was best described by a quadratic polynomial function with a correlation coefficient (R^2^) of 0.945 (Fig. 3). Furthermore, the correlations of the respective donors were analyzed individually and displayed a markedly lower deviation (Fig. 3A). However, it is clearly shown in Fig. 3A that the measured endotoxin levels of sera spiked with 50 ng/ml LPS are strongly donor dependent. The mean calculated residual standard deviation, which represents the dispersion of the measured values around the regression line (Sy.x), in the measured concentration range between 0 and 50 EU/ml is 4.44 EU/ml in the pool and between 0.53 to 3.89 EU/ml in the donor-specific evaluation (Fig. 3B,C).

### Influence of clotting on the recovery of LPS

**Figure 4:**
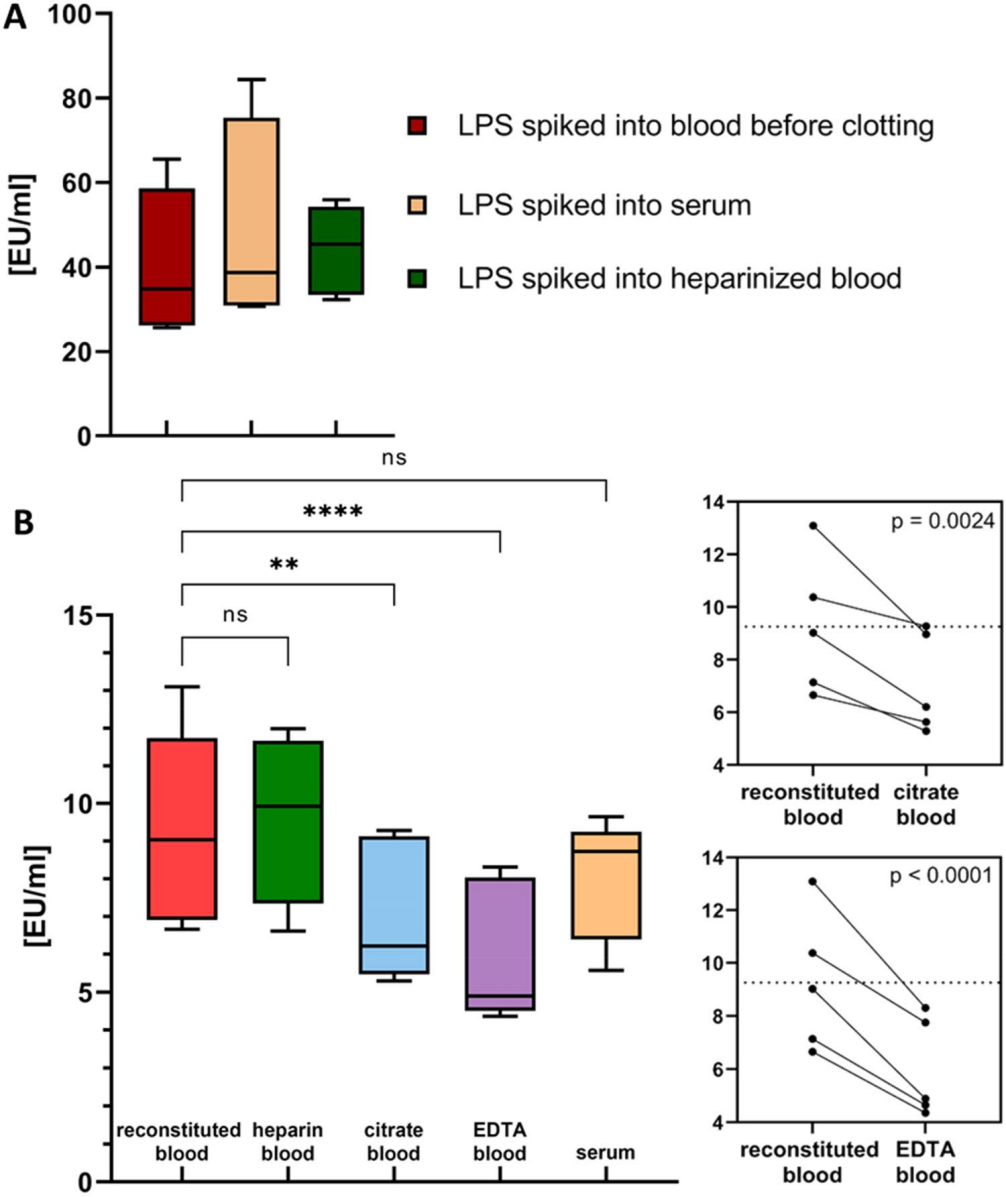
Influence of blood coagulation and different anticoagulants on LPS recovery. (**A**) Influence of blood coagulation. From each donor the LPS concentration was measured after spiking LPS to the whole blood before clotting, to heparin anticoagulated whole blood and serum. The mean LPS values ± SD are shown (n = 5). (**B**) Influence of different anticoagulants. “Reconstituted blood”, spiked with LPS was transferred to Vacuettes containing heparin, citrate, or EDTA and LPS was quantified using the improved protocol. Serum without blood cells served as control. Mean values ± SD are indicated (n = 5). The respective paired t-test plots show that each individual endotoxin measurement is significantly lower in EDTA and citrated blood compared to “reconstituted blood”.

For the analysis of clinical samples, the use of serum *vs.* anticoagulated plasma is a frequent matter of discussion. As a prerequisite for the use of serum samples, a potential loss of LPS during clot formation has to be excluded, since previous studies have suggested that clotting may lead to LPS adsorption^23^. To further investigate this issue, we added LPS to non-anticoagulated blood immediately after blood collection, to heparinized blood, as well as to freshly collected serum. LPS quantification in all three samples yielded comparable results, indicating that blood clotting during the generation of serum does not affect LPS levels (Fig. 4A).

### Influence of anticoagulants on the recovery of LPS

To simulate the collection of LPS-containing whole blood into tubes containing different anticoagulants, we used “reconstituted blood” prepared by incubation of LPS in human serum and subsequent addition of washed blood cells from the same donor. This “reconstituted blood” was transferred into Vacuettes containing heparin, EDTA, citrate, or no anticoagulant. Serum without blood cells served as control (Fig. 4B). The recovery of LPS was not significantly (p ≤ 0.05) different for heparinized blood (9.59 ± 2.24 EU/ml) and “reconstituted blood” (9.26 ± 2.61 EU/ml), while significantly lower LPS recovery was obtained for citrate tubes (7.08 ± 1.90 EU/ml) and EDTA tubes (6.00 ± 162 EU/ml).

### Masking effect in serum

**Figure 5:**
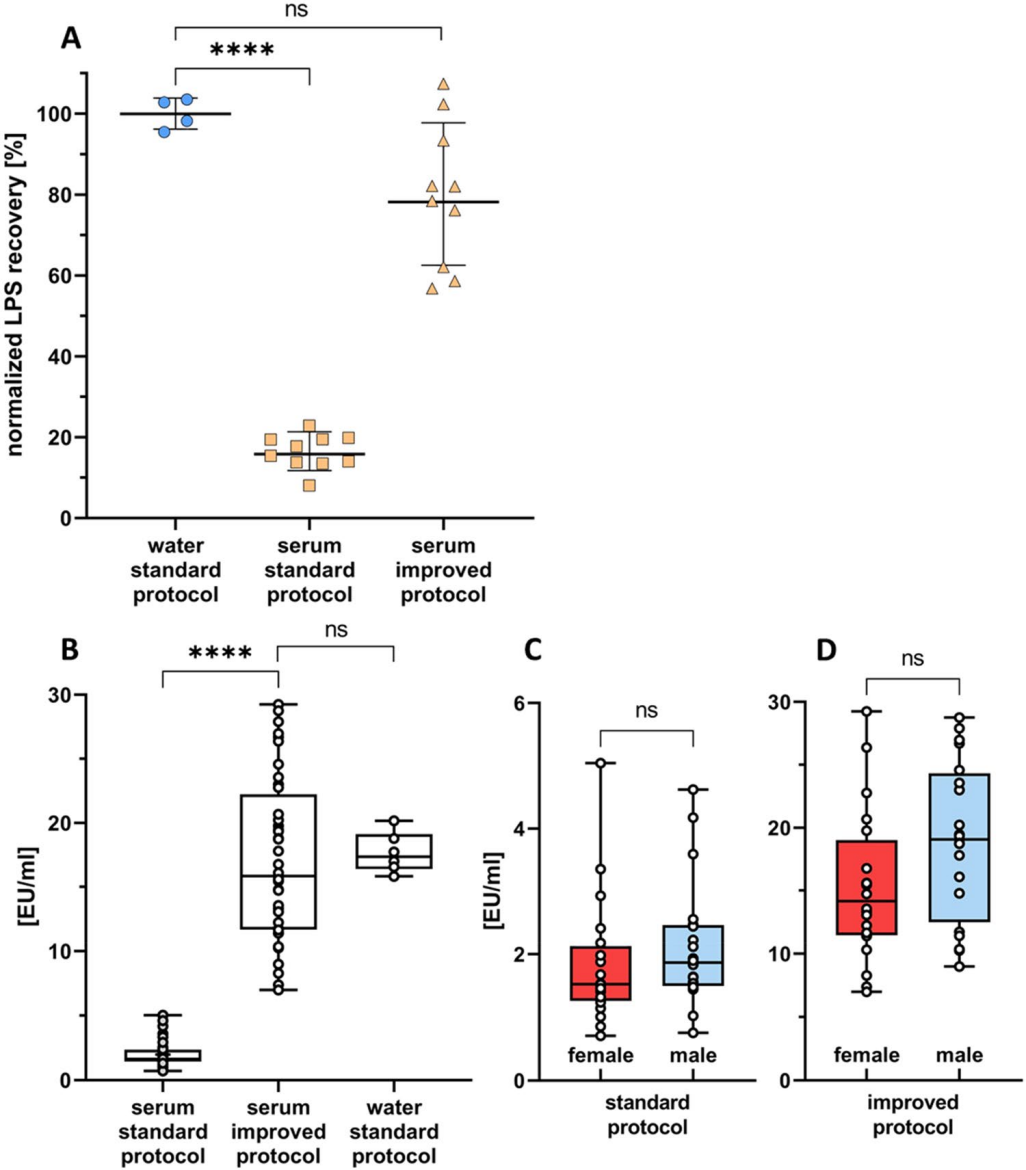
Recovery of LPS using the standard *vs.* improved protocol of the LAL assay. (**A**) Serum from different donors (n = 10) and aliquots of pyrogen-free water (n = 4) were spiked with a BRP standard (25 EU/ml). Serum samples were measured using the standard and the improved protocol, and normalized LPS recovery was calculated as a percentage compared to water samples. Individual results as well as mean values ± SD are indicated (n = 10). (**B**) LPS recovery in 40 donors using the improved and standard protocol. Serum samples from 40 volunteer donors (20 females and 20 males) were spiked with 50 ng/ml LPS and analyzed using the standard and the improved protocol and compared to LPS spiked water samples (n = 6). (C,D) LPS recovery separated by gender. The values are presented as boxplots with the median ± SD (n = 40).

According to the pharmacopoeia, the test matrix is considered free of interfering factors if the measured LPS concentration is within 50 – 200% of the added endotoxin standard (BRP). The LPS values measured in serum samples were compared to mean LPS values in water to determine recovery rate. This rate, expressed as a percentage, is a normalized recovery, assuming 100% LPS recovery in water. We found that the recovery of spiked LPS in human serum using the standard protocol was only 16 ± 4%, as compared to the recovery in water. With the improved protocol, a LPS recovery of 80 ± 18% was achieved, and the recovery was not significantly different (p = 0.08) between water and serum, indicating that the improved protocol eliminates most of the factors interfering with the LAL assay in serum samples (Fig. 5A).

### LPS recovery in serum samples compared between standard and improved protocol

To compare the LPS recovery in human serum with the standard protocol *vs.* the improved protocol, we used spiked sera from 40 healthy donors (20 females and 20 males) with an average age of 38 ± 19 years. The average endotoxin values measured in the serum samples using the standard protocol were 1.98 ± 0.99 EU/ml, whereas the improved protocol showed 17.12 ± 6.35 EU/ml. The comparison group of water samples, treated identically to the serum samples, displayed an endotoxin value of 17.69 ± 1.58 EU/ml using the standard protocol. The improved protocol yielded an increase of the normalized recovery rate from 11 ± 6 to 97 ± 36% in the serum samples. On average, the LPS levels in sera measured with the improved protocol were average 8.7 times higher than those measured using the standard protocol (Fig. 5B). We observed a trend towards higher endotoxin levels in males than in females (Fig. 5C,D), but the difference did not reach statistical significance.

## Discussion

The reliable detection of LPS in clinical samples is hampered by the presence of various factors that either directly interfere with the LAL assay or mask endotoxin^24^. While it is well established that human plasma samples can neutralize endotoxins^25,26^, the mechanisms underlying endotoxin masking are not yet fully elucidated. One potential mechanism of LPS inactivation is described by the endotoxin-lipoprotein hypothesis^27–29^, which states that LPS is bound and neutralized by lipoproteins, particularly by high density lipoprotein (HDL). HDL serves as a transport vehicle carrying LPS to the liver, where it is disposed into the intestine via the bile. Other authors have demonstrated neutralization of endotoxin lipoprotein-free protein plasma fractions, as well^20^.

We have previously demonstrated that heparin affects LPS activity in human whole blood^20^, and that both, the quantification of endotoxin using the LAL assay and the immunostimulatory properties of LPS in whole blood were strongly increased in the presence of heparin. These observations led us to hypothesize that the polyanion heparin binds to cationic plasma components, which are partly responsible for LPS neutralization in human plasma. We thus developed an improved protocol for the pretreatment of human serum and plasma samples to optimize the LPS recovery in the LAL assay. This improved protocol is based on using a sample diluent containing heparin (10 IU/ml) and magnesium (5 mM), which is an essential component in the LAL cascade. A tenfold dilution of serum samples in this solution resulted in beneficial LPS recovery. The addition of calcium or of a combination of calcium and magnesium at a 2:1 ratio, which corresponds to the physiological ratio in human blood, negatively impacted LPS recovery. This might be due to the fact that the horseshoe crab, from which the LAL reagent is derived, has evolved in an environment with a Mg:Ca ratio of 5:1^30^.

The improved protocol shows linearity in serum samples containing between 0 and 50 ng/ml LPS. However, it is evident that the measured levels of endotoxin in serum spiked with LPS exhibit significant variation among donors. According to the pharmacopoeia, LPS recovery in the test matrix must be between 50 to 200% as compared to measurement in water for the results to be valid. Testing for “masking effect” must be performed for each matrix prior to the LAL assay. In serum and plasma samples, the required recovery rate is not achieved using the standard protocol recommended by the manufacturer, which is likely why LAL assays have not yet found entry into the field of medical diagnostics. However, using the improved protocol, the required LPS recovery rate in serum samples was reproducibly achieved, suggesting that approval of this method for measuring endotoxin levels in blood samples is possible.

We further addressed the question whether serum or plasma samples should be used for LAL testing, since removal of the clot during generation of serum might be associated with adsorption of LPS. Our data did, however, not provide any indication for a loss of LPS during clotting, and we obtained comparable results for serum and plasma samples. These findings contradict previous reports by Armstrong et al.^23^, who observed LPS adsorption to fibrin. It is conceivable that the adsorption of LPS to fibrin fibers and platelet aggregates was due to the use of FITC-labeled LPS in this previous study since the coupling of several FITC molecules per molecule of LPS may have altered its characteristics.

When assessing the influence of the anticoagulant used during blood collection, we found that next to serum tubes, plasma anticoagulated with heparin yielded the highest LPS recovery, whereas citrate and EDTA led to significantly lower endotoxin levels. LPS aggregates exist as micelles stabilized by divalent ions, and their complexation by EDTA or citrate results in destabilization of the LPS micelles, which may result in binding of LPS monomers to plasma proteins. Although the diluent containing 5 mM Mg^++^ is sufficient to stabilize the LPS micelles and act as cofactor for the LAL cascade, this process does not seem to be completely reversible.

To further evaluate the improved protocol for sample pretreatment, we used sera from 40 healthy donors spiked with 50 ng/ml LPS and were able to confirm that endotoxin levels were more than 8 times higher using the improved protocol as compared to the standard protocol. This clearly demonstrates that the heparin-containing diluent inactivates endotoxin-neutralizing plasma components, leading to a higher endotoxin recovery. We also observed a trend towards a higher LPS recovery in male serum than in female serum, suggesting that females have a higher endotoxin neutralizing capacity than males. Similar findings were obtained in a clinical study by Champion et al*.*^31^, where significantly higher endotoxin neutralization in plasma was measured in women than in men. Accordingly, women have a significantly better sepsis prognosis than men, significantly lower in-hospital mortality, and a better response to traumatic injury^32–34^.

Our data show that a large proportion of LPS spiked into plasma of healthy donors is neutralized. The question arises whether this endotoxin-neutralizing capacity is reduced in patients suffering from sepsis and whether the ratio of free LPS and total LPS (measured after heparin addition) might be useful as a biomarker. It remains to be elucidated which plasma components/AMPs are mainly responsible for the neutralization of LPS, and their identification might support the development of new, particularly blood-compatible antimicrobial compounds.

To validate the improved protocol for LAL-based endotoxin quantification for clinical use, further studies with serum samples from patients suffering from sepsis, liver failure, or other inflammatory pathologies caused by Gram-negative bacteria will be required.

## Materials and methods

### Human whole blood

Human whole blood was obtained from healthy volunteer donors. All blood donations were approved by the Ethics Committee of the University for Continuing Education Krems (EK GZ 13/2015–2018). Written informed consent was obtained from all blood donors. All experiments were conducted in accordance with the guidelines of the Declaration of Helsinki of the World Medical Association. Blood samples were collected by trained personnel using the Vacuette^®^ safety blood collection set. Depending on the specific testing requirements, the following blood collection tubes were utilized: K3E K3EDTA, NH Sodium Heparin, 9NC Coagulation Trisodium Citrate 3.2% and CAT Serum. All materials were purchased from Greiner Bio-One (Kremsmünster, Austria). Serum or plasma was obtained by centrifugation (10 min, 3500 rpm, room temperature).

### Standard LAL assay protocol for plasma and serum samples

LPS was quantified using the kinetic chromogenic Limulus amebocyte lysate (LAL) assay (Charles River, Wilmington, MA) according to the instructions of the manufacturer. Samples were diluted 1:10 with pyrogen-free water in pyrogen-free tubes (Charles River) and incubated at 70 °C for 15 min to denature plasma proteins and proteases interfering with the LAL assay^35,36^. Aliquots of 100 µL of each standard and sample were transferred into a 96-well flat-bottomed microplate (Charles River) in duplicate and incubated at 37 °C for 10 min in a microplate reader (Tecan Sunrise, r-biopharm, Darmstadt, Germany). Following the addition of 100 µL LAL reagent per well, the kinetic color development at 405 nm was recorded, and the LPS concentration in the individual samples was calculated from the standard curve.

### Improved protocol for the LAL assay

Plasma or serum samples were diluted 1:10 in pyrogen-free water containing 5 mM Mg^++^ (Merck, Darmstadt, Germany) and 10 IU/ml heparin (Gilvasan Pharma GmbH, Vienna, Austria) and incubated for three hours at room temperature. During incubation, endotoxin-inactivating cationic plasma components bind to the polyanion heparin and are inactivated. All subsequent steps were performed as described in the standard protocol above. The linearity of the improved protocol was tested in serum from five healthy donors spiked with 50, 25, 12.5, 3.13, and 0.78 ng/ml LPS.

### Endotoxin neutralizing capacity

The recovery of endotoxin was determined in a dilution series of human serum spiked with LPS. Serum from six healthy donors was collected and diluted with physiological saline containing 1.2 mM Ca^++^ and 0.6 mM Mg^++^ (Pharmacy Bad Ischl, Austria) to obtain a dilution series containing 0, 1, 5, 10, 20, 40, 60, 80, and 100% serum. All samples were spiked with 50 ng/ml LPS from *E. coli* O55:B5 (Sigma Aldrich, St. Louis, MO), incubated overnight at room temperature with gentle rolling, and analyzed using the standard LAL assay protocol.

### Influence of heparin concentration on endotoxin recovery

To determine the optimal heparin concentration for suppressing the endotoxin neutralizing effect of plasma, serum was obtained from six healthy donors, spiked with increasing amounts of heparin (0, 5, 10, 20, 30, 40, 50, 75 and 100 IU/ml) and with 50 ng/ml LPS from *E. coli,* incubated overnight at room temperature with gentle rolling, and analyzed using the standard LAL assay protocol. To better assess the recovery of measured endotoxin levels in serum, a comparative measurement was conducted in endotoxin-free water (n = 6) under the same conditions as the serum samples. With the obtained endotoxin values the normalized LPS recovery (%) was calculated as followed:

$$normalized\,LPS\,recovery\,\left[\%\right]= \frac{mean\,measured\,LPS\,value\,in\,water\, [EU/ml]}{individual\,measured\,LPS\,value\,in\,serum [EU/ml]} \times 100$$

### Influence of exposure time to heparin on endotoxin recovery

Serum from six different donors was incubated overnight at room temperature with 50 ng/ml LPS from *E. coli* with gentle rolling. Aliquots of 980 µl LPS-spiked serum were incubated with 20 µl heparin solution (5000 IU/ml) for 10 min, 30 min, 1 h, 2 h, 4 h, 6 h, and 24 h. After incubation, all samples were diluted 1:10 in pyrogen-free water and analyzed using the standard LAL assay protocol. This direct addition of heparin to the LPS-spiked serum was compared to the use of a heparin-containing diluent, where the LPS-spiked samples were diluted 1:10 in pyrogen-free water containing 10 IU/ml heparin.

### Influence of Ca^++^ and Mg^++^ on endotoxin recovery

To assess the influence of Ca^++^ or Mg^++^ on endotoxin recovery in serum, diluents containing 0 mM, 1 mM, 5 mM, 10 mM, or 15 mM Ca^++^ or Mg^++^ in pyrogen-free water, spiked with 10 IU/ml heparin were prepared. In addition, a diluent containing Ca^++^ and Mg^++^ at a 2:1 ratio was prepared, reflecting the ratio of divalent ions in human blood. Serum from six different donors was incubated with 50 ng/ml LPS from *E. coli* overnight with gentle rolling. The LPS-spiked sera were diluted 1:10 with the diluent described above, incubated 15 min at 70 °C and endotoxin quantification was performed using the LAL assay.

### Influence of clot formation on LPS recovery in serum samples

To determine whether the formation of blood clots during the collection of serum affects LPS recovery, e.g., by binding of LPS to the clot, whole blood without anticoagulant and heparin-anticoagulated blood was drawn from five healthy donors. Immediately after blood collection, an aliquot of the non-anticoagulated blood was spiked with 30 ng/ml LPS (*E. coli*). When clotting was completed, the serum was collected by centrifugation (3500 g, 10 min), and endotoxin was quantified using the improved LAL assay protocol (see above). The spike concentration of 30 ng/ml LPS corresponds to a serum level of 50 ng/ml LPS at a hematocrit of 40% assuming that LPS only distributes in plasma. For comparison, 50 ng/ml of LPS was added directly to serum and analyzed as described above.

### Influence of different anticoagulants on LPS recovery

Separate tests were conducted to identify the most effective anticoagulant for enhancing recovery during blood sampling for clinical endotoxin quantification.

Whole blood without anticoagulant and citrate-anticoagulated blood was drawn. The serum was spiked with 50 ng/ml LPS from *E. coli*. Blood cells in the whole blood samples anticoagulated with citrate were pelleted by centrifugation and washed three times with physiological saline. The LPS-spiked serum and blood cells from the same donor were mixed at a 1:1 ratio, to obtain “reconstituted blood” with a hematocrit of about 40%. This “reconstituted blood” was incubated overnight at 37 °C with gentle shaking and then transferred to Vacuettes containing, citrate, EDTA, or no additives and subsequently incubated for an additional hour under the same conditions (Supplementary Information S1). Then, the samples were centrifuged and LPS was quantified using the improved protocol. LPS-spiked serum was used as control.

### Determination of masking effect in serum

In order to determine the “masking effect” of serum with regard to endotoxin recovery using the LAL test, BRP standard was added to fresh serum samples and measured immediately afterwards using the standard and improved protocol. Sera from ten donors were spiked with 25 EU/ml of an international endotoxin standard (BRP, Sigma-Aldrich, St. Louis, USA) and endotoxin was quantified immediately using the standard and the improved protocol. Pyrogen-free spiked water with 25 EU/ml endotoxin standard BRP served as reference. The endotoxin concentration obtained using the two protocols was compared to the endotoxin concentration measured in aqueous solution, and the normalized LPS recovery (%) was calculated.

### LPS recovery in serum samples compared between standard and improved protocol

Serum samples were obtained from 20 male and 20 female volunteer donors and stored at − 80 °C. Prior to analysis, they were spiked with 50 ng/ml LPS from *E. coli* and incubated overnight at room temperature. On the next day, endotoxin measurement was performed using both, the standard protocol, and the improved protocol. For better comparability, 50 ng/ml LPS was spiked into endotoxin-free water, incubated overnight and the endotoxin concentration measured using the standard protocol (n = 6).

### Statistical analysis

Calculations of means, standard deviations (SD) and all other statistical tests were carried out using GraphPad Prism 9.3.1 (GraphPad Software, Boston, MA). The Kolmogorov–Smirnov Test was applied to check for normal distribution. Normal distributed data were compared using the t test. For non-normally distributed data, the Mann–Whitney Rank Sum Test was used. Significances are given in the manuscript as follows: ns p > 0.05, *p ≤ 0.05, **p ≤ 0.01, ***p ≤ 0.001 and ****p ≤ 0.0001.

### Supplementary Information

Supplementary Figure S1.

Supplementary Information.

## Data Availability

All data generated or analyzed during this study are included in this published article (supplementary Information S2).
