# Supplementary figures and images for: Heparin enables the reliable detection of endotoxin in human serum samples using the Limulus amebocyte lysate assay
Source: Sci Rep. 2024 Jan 29;14:2410. doi: 10.1038/s41598-024-52735-8 (PMC10825173; doi:10.1038/s41598-024-52735-8)

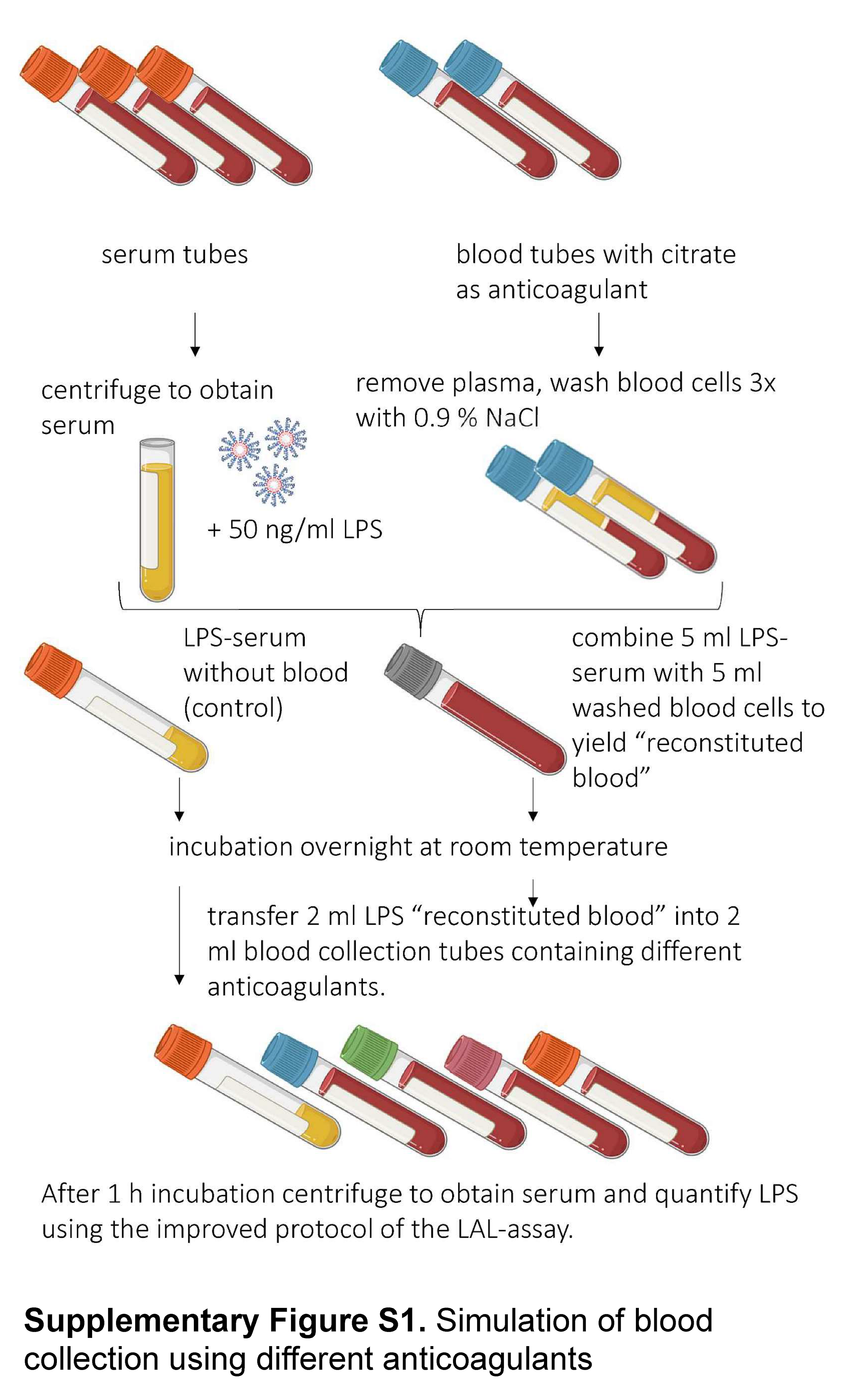

Supplement: Supplementary file 1 — Supplementary Figure S1. [file 41598_2024_52735_MOESM1_ESM.tif]
