# Supplementary material for: Heparin enables the reliable detection of endotoxin in human serum samples using the Limulus amebocyte lysate assay
Source: Sci Rep. 2024 Jan 29;14:2410. doi: 10.1038/s41598-024-52735-8 (PMC10825173; doi:10.1038/s41598-024-52735-8)
Supplement: Supplementary file 2 — Supplementary Information. [file 41598_2024_52735_MOESM2_ESM.pdf]

Supplementary information S2. All datasets generated during and/or analyzed during the current study are given in following tables.

Figure 1. **Endotoxin neutralizing capacity.**

Serum [%]	Donor 1	Donor 2	Donor 3	Donor 4	Donor 5	Donor 6	Unit
100	5.904	5.196	3.190	5.163	1.031	2.347	[EU/ml]
80	6.337	6.364	4.207	6.150	1.147	3.053	[EU/ml]
60	7.880	8.516	5.693	7.495	1.418	3.497	[EU/ml]
40	11.934	12.026	9.392	11.510	2.257	5.791	[EU/ml]
20	24.762	25.837	20.917	24.023	7.602	14.562	[EU/ml]
10	46.157	44.029	36.737	39.201	21.816	33.886	[EU/ml]
5	75.904	76.967	57.288	63.750	52.777	52.101	[EU/ml]
1	163.662	155.389	101.136	131.209	96.252	92.529	[EU/ml]
0	120.277	101.195	144.186	153.165	140.247	136.285	[EU/ml]

Figure 2. A **LPS recovery depending on heparin concentration.**

Heparin [IU/ml]	Donor 1	Donor 2	Donor 3	Donor 4	Donor 5	Donor 6	Unit
0	2.283	7.917	1.771	2.174	2.651	5.891	[EU/ml]
5	17.053	26.261	6.414	4.338	8.011	11.186	[EU/ml]
10	16.685	31.513	10.315	5.263	9.112	14.512	[EU/ml]
20	23.257	34.568	11.693	6.368	11.377	12.768	[EU/ml]
30	24.632	38.32	13.16	7.255	13.955	11.748	[EU/ml]
40	24.812	38.842	15.925	8.202	14.514	12.006	[EU/ml]
50	28.832	41.819	17.226	10.158	16.231	13.566	[EU/ml]
75	35.107	52.034	20.336	11.334	15.846	17.649	[EU/ml]
100	42.812	56.923	23.081	11.770	16.827	20.508	[EU/ml]

LPS in water (n=6)	
25.553	EU/ml

Figure 2. B **Influence of exposure time to heparin.**

	Donor 1	Donor 2	Donor 3	Donor 4	Donor 5	Donor 6	
time [min]	heparin containing diluent						Unit
0.1	11.510	3.464	1.464	3.842	4.534	2.037	[EU/ml]
10	29.872	11.569	7.665	15.297	6.048	5.514	[EU/ml]
30	28.625	11.530	7.512	14.389	7.362	5.439	[EU/ml]
60	29.192	11.056	7.523	15.042	11.145	8.001	[EU/ml]
120	32.368	11.491	7.390	18.827	17.958	6.157	[EU/ml]
180	38.074	11.403	7.118	20.501	18.396	7.042	[EU/ml]
240	37.989	11.641	6.998	21.131	15.423	6.686	[EU/ml]
360	35.768	12.425	7.193	19.143	17.078	8.221	[EU/ml]
1440	37.76	12.380	8.609	26.133	26.699	12.189	[EU/ml]
time [min]	heparin directly to serum						Unit
0.1	5.730	2.824	1.628	4.272	4.105	1.256	[EU/ml]
10	15.769	10.062	7.724	15.303	13.379	6.399	[EU/ml]
30	14.75	9.880	7.587	14.46	12.838	6.494	[EU/ml]
60	15.46	9.418	7.571	15.454	12.503	6.595	[EU/ml]
120	16.966	10.677	7.463	17.186	13.453	6.365	[EU/ml]
180	20.165	9.703	7.419	20.813	13.303	6.294	[EU/ml]
240	20.043	11.166	7.319	20.048	15.065	6.055	[EU/ml]
360	18.943	11.499	7.513	19.461	16.037	6.504	[EU/ml]
1440	20.600	12.107	10.567	20.802	15.253	8.326	[EU/ml]

Figure 2. C **Influence of divalent cations on LPS recovery.**

	Donor 1	Donor 2	Donor 3	Donor 4	Donor 5	Donor 6	
[mM]	Mg^{++}						Unit
0	15.227	9.226	5.184	23.643	16.861	14.177	[EU/ml]
1	16.145	10.146	6.212	22.596	18.468	15.181	[EU/ml]
5	18.933	11.375	7.643	24.751	22.20	17.492	[EU/ml]
10	20.978	10.298	7.595	25.346	19.132	18.769	[EU/ml]
15	22.004	9.746	8.134	28.138	19.476	18.284	[EU/ml]
[mM]	Ca^{++}						Unit
0	15.227	9.226	5.184	23.643	16.861	14.177	[EU/ml]
1	12.716	5.875	5.399	16.989	11.805	13.44	[EU/ml]
5	7.699	4.109	2.258	7.361	5.342	4.699	[EU/ml]
10	3.934	2.355	2.093	4.779	3.365	2.775	[EU/ml]
15	2.464	1.572	1.258	3.571	2.597	1.549	[EU/ml]
[mM]	Ca^{++}/Mg^{++}(2:1)						Unit
0	15.227	9.226	5.184	23.643	16.861	14.177	[EU/ml]
1	12.918	7.101	5.193	16.263	12.334	10.145	[EU/ml]
5	9.753	4.737	2.853	7.294	7.010	5.997	[EU/ml]
10	4.759	2.853	1.701	3.255	2.881	2.389	[EU/ml]
15	2.648	2.494	1.641	2.303	2.080	1.880	[EU/ml]

Figure 3. A **Linearity of the improved protocol.**

Correlation between spiked and measured LPS values in human serum using the improved protocol for LAL analysis.

spiked LPS [ng/ml]	Donor 1	Donor 2	Donor 3	Donor 4	Donor 5	Unit
0.000	0.000	0.000	0.000	0.000	0.000	[EU/ml]
0.78125	5.303	1.256	3.448	2.002	1.506	[EU/ml]
3.125	7.308	9.746	8.897	6.863	9.128	[EU/ml]
12.500	17.991	12.959	24.527	22.672	18.37	[EU/ml]
25.000	27.974	21.611	37.611	34.98	30.96	[EU/ml]
50.000	42.54	42.574	59.094	54.174	50.86	[EU/ml]

Figure 4. A **Influence of blood coagulation and different anticoagulants on LPS recovery.**

	LPS spiked into blood before clotting	LPS spiked into serum	LPS spiked into heparinized blood	Unit
Donor 1	65.541	38.697	55.934	[EU/ml]
Donor 2	25.684	31.148	45.406	[EU/ml]
Donor 3	51.73	66.301	52.708	[EU/ml]
Donor 4	34.834	84.401	34.496	[EU/ml]
Donor 5	26.601	30.656	32.276	[EU/ml]

Figure 4. B **Influence of different anticoagulants.**

	reconstituted blood	heparin blood	citrate blood	EDTA blood	serum	Unit
Donor 1	7.147	6.613	5.295	4.656	5.568	[EU/ml]
Donor 2	13.099	11.987	8.973	8.309	8.847	[EU/ml]
Donor 3	9.033	9.921	6.214	4.893	8.723	[EU/ml]
Donor 4	6.662	8.077	5.637	4.363	7.204	[EU/ml]
Donor 5	10.380	11.350	9.277	7.759	9.646	[EU/ml]

Figure 5. A **Normalized recovery of LPS using the standard *vs.* improved protocols of the LAL assay.**

	water	serum standard protocol	serum improved protocol	Unit
Donor 1	102.205636	13.76411058	58.62414423	[EU/ml]
Donor 2	102.896965	13.47952736	56.79864696	[EU/ml]
Donor 3	94.8973996	8.04468188	62.08078924	[EU/ml]
Donor 4	98.1972247	19.80282779	102.3180808	[EU/ml]
Donor 5	-	17.74827573	76.11560094	[EU/ml]
Donor 6	-	13.97928327	82.12655393	[EU/ml]
Donor 7	-	22.86383271	93.38494393	[EU/ml]
Donor 8	-	19.46271613	107.4336377	[EU/ml]
Donor 9	-	19.43495191	82.01549706	[EU/ml]
Donor 10	-	15.4160815	78.4130899	[EU/ml]

Figure 5. B

LPS recovery in 40 donors using the improved and standard protocols.

		standard protocol	improved protocol	water standard protocol	Unit
Donor 1	Donor1	1.010	7.400	20.1656	[EU/ml]
Donor 2	Donor2	1.409	13.338	17.7395	[EU/ml]
Donor 3	Donor3	3.597	23.000	15.8378	[EU/ml]
Donor 4	Donor4	1.534	13.574	17.0083	[EU/ml]
Donor 5	Donor5	4.175	26.706	16.5725	[EU/ml]
Donor 6	Donor6	1.882	26.388	18.8002	[EU/ml]
Donor 7	Donor7	2.470	24.575		[EU/ml]
Donor 8	Donor8	1.835	11.730		[EU/ml]
Donor 9	Donor9	5.044	29.244		[EU/ml]
Donor 10	Donor10	1.982	15.477		[EU/ml]
Donor 11	Donor11	3.357	19.777		[EU/ml]
Donor 12	Donor12	1.147	12.247		[EU/ml]
Donor 13	Donor13	1.531	15.614		[EU/ml]
Donor 14	Donor14	1.559	10.263		[EU/ml]
Donor 15	Donor15	1.498	10.323		[EU/ml]
Donor 16	Donor16	2.444	19.472		[EU/ml]
Donor 17	Donor17	0.858	6.995		[EU/ml]
Donor 18	Donor18	2.179	22.772		[EU/ml]
Donor 19	Donor19	1.932	19.364		[EU/ml]
Donor 20	Donor20	1.240	8.294		[EU/ml]
Donor 21	Donor21	1.642	8.991		[EU/ml]
Donor 22	Donor22	1.634	11.420		[EU/ml]
Donor 23	Donor23	1.900	27.899		[EU/ml]
Donor 24	Donor24	2.226	26.986		[EU/ml]
Donor 25	Donor25	1.445	18.793		[EU/ml]
Donor 26	Donor26	1.458	18.733		[EU/ml]
Donor 27	Donor27	1.540	16.109		[EU/ml]
Donor 28	Donor28	2.556	14.800		[EU/ml]
Donor 29	Donor29	1.481	20.223		[EU/ml]
Donor 30	Donor30	0.755	10.362		[EU/ml]
Donor 31	Donor31	2.121	23.56		[EU/ml]
Donor 32	Donor32	1.0252	17.811		[EU/ml]
Donor 33	Donor33	4.623	28.751		[EU/ml]
Donor 34	Donor34	1.524	15.592		[EU/ml]
Donor 35	Donor35	0.7099	11.402		[EU/ml]
Donor 36	Donor36	1.462	11.668		[EU/ml]
Donor 37	Donor37	2.413	16.784		[EU/ml]
Donor 38	Donor38	2.930	20.683		[EU/ml]
Donor 39	Donor39	1.678	14.751		[EU/ml]
Donor 40	Donor40	1.323	13.080		[EU/ml]

Figure 5. C-D **LPS recovery in 40 donors using the improved and standard protocols separated by gender.**

standard protocol		improved protocol		
female	male	female	male	Unit
7.400	23,00	1.010	3.597	[EU/ml]
13.338	26.706	1.409	4.175	[EU/ml]
13.574	24.575	1.534	2.470	[EU/ml]
26.388	11.730	1.882	1.835	[EU/ml]
29.244	10.263	5.044	1.559	[EU/ml]
15.477	19.472	1.982	2.444	[EU/ml]
19.777	19.364	3.357	1.932	[EU/ml]
12.247	8.991	1.147	1.642	[EU/ml]
15.614	11.420	1.531	1.634	[EU/ml]
10.323	27.899	1.498	1.900	[EU/ml]
6.995	26.986	0.858	2.226	[EU/ml]
22.772	18.793	2.179	1.445	[EU/ml]
8.294	18.733	1.240	1.458	[EU/ml]
15.592	16.109	1.524	1.540	[EU/ml]
11.402	14.800	0.7099	2.556	[EU/ml]
11.668	20.223	1.462	1.481	[EU/ml]
16.784	10.362	2.413	0.755	[EU/ml]
20.683	23.56	23.560	2.121	[EU/ml]
14.751	17.811	1.678	1.0252	[EU/ml]
13.080	28.751	1.323	4.623	[EU/ml]
